# Heterogeneity in the association between social support and mental distress in old-age retirees – a computational approach using longitudinal cohort data

**DOI:** 10.1186/s12877-024-05384-5

**Published:** 2024-10-08

**Authors:** Jere Lahelma, Eero Lahelma, Mikko Laaksonen, Susan Kuivalainen, Mikko Koivisto, Tea Lallukka

**Affiliations:** 1 Department of Computer Science, University of Helsinki, Helsinki, Finland; 2 Finnish Centre for Pensions, Helsinki, Finland; 3 Department of Public Health, University of Helsinki, Helsinki, Finland

**Keywords:** Mental distress, Social support, Longitudinal study, Model-based recursive partitioning, Old-age retiree, DASS-21, Decision tree, Subgroup analysis

## Abstract

**Background:**

Mental distress among retirees and older people is a severe public health challenge, and information on new risk groups is needed. This study aims to identify subgroups of old-age retirees with varying associations between low social support and mental distress by applying model-based recursive partitioning (MOB).

**Methods:**

We used the Helsinki Health Study follow-up survey data of old-age retired former municipal sector employees of the City of Helsinki, Finland. Phase 1 data were collected in 2000–2002, when all participants were employed, Phase 2 in 2007, Phase 3 in 2012, Phase 4 in 2017, and Phase 5 in 2022 (*n* = 4,466, 81% women). Social support and covariates were measured at each Phase 1–5 and the outcome, mental distress (Depression Anxiety Stress Scales [DASS-21]) was measured at a single occasion, during Phase 5. The three subscales and the common factor of general distress were analysed separately. An approach rooted in computational statistics was used to investigate risk factor heterogeneity in the association of low social support and mental distress. MOB combines decision trees with regression analysis to identify subgroups with the most significant heterogeneity among risk factors.

**Results:**

Median (IQR) general distress score from DASS-21 was 5.7 (3.0, 9.0), while Social Support Questionnaire number-score (SSQN) was 1.5 (1.15, 2.05). The primary effect modifier for the association between social support and general distress was education (*p* < 0.001). Those with high education had a different association of low social support and general distress than those with low or medium education. Additionally, the subgroup with low and medium education had a significant effect modification for age (*p* = 0.01). For the association between low social support and depressive symptoms, the moderating effect of education was dependent on gender, as men with medium–high education had the weakest association, while for women with medium–high education the association was strongest.

**Conclusions:**

Our results suggest that stratification by sociodemographic variables is justifiable when investigating risk factors of mental distress in old-age retirees. The incongruent association of low social support and depressive symptoms in men with medium–high education compared to women with medium–high education is a promising target for confirmatory research.

**Supplementary Information:**

The online version contains supplementary material available at 10.1186/s12877-024-05384-5.

## Background

Population ageing is challenging health and social care systems due to increasing the health burden in older people [1]. Among the health problems, mental distress is increasingly common.

The OECD estimates the costs of mental health problems at around 4% of GDP across the EU, which translates to around 600 billion euros per year [2]. At age 75 + in 2014, 12.4% of women and 6.5% of men in the EU had chronic depression [3]. Therefore, investigating the determinants of mental distress among retirees is of great importance. To improve mental health promotion and prevention, it is necessary to have a better understanding of potential subgroups and the heterogeneity of risk factors. Many older people live alone, and loneliness and isolation are linked to adverse health outcomes, including even mortality [4, 5]. The importance of social risk factors for the mental health of older people is particularly highlighted [6], and they have been linked to both depressive and anxiety symptoms [7].

Social support is an important risk factor preceding depression and anxiety disorders in older adults [8, 9]. Low social support has been shown to form a multifactorial construct in relation to depression in older adults, such as perceived social support, structure of the social network or as tangible help [10]. Previous studies have shown that perceived social support is especially robust determinant of depression in older adults [10]. Additionally, low social support has been shown to be associated with anxiety disorders in older adults [8, 9] and thus further examination of its role in older people’s mental health is needed.

Previous studies have also found effect modifications for the risk factors for mental distress. On the one hand, among older men with physical limitations who desire greater independence, social support may have a weaker contribution to depressive symptoms [8]. On the other hand, in a cross-sectional, nationally representative Swedish sample, older men in the high-risk group showed a stronger association between low social support and depressive symptoms than women [11]. At higher levels of education, the association between social isolation and mental health symptoms has been shown to be stronger than at lower levels of education, indicating that education has a plausible moderating effect on the social risk factors for mental distress [12]. Older adults who are not married or cohabiting have shown higher rates of anxiety disorders even when adjusting for confounders [13]. There is, however, conflicting evidence between longitudinal and cross-sectional studies about potential gender differences in the associations of low social support from the spouse and depression. Longitudinal studies have shown evidence for the protective contribution of spousal support, especially for men, while cross-sectional studies have highlighted the importance for men and women, implicating potential benefits of further exploratory research on the topic [14]. Psychiatric frameworks for mood disorders emphasize the multiplicative effects of risk factors, demonstrating the advantage of using exploratory methods that focus on finding new potential interaction effects [15]. Along with these previously known effect modifications, using sociodemographic variables like gender, marital status, age, and education as effect modifiers enables the arrangement of actionable subpopulations [16]. In prior studies with the HHS (Helsinki Health Study) cohort, women have shown higher rates of mental health symptoms [17]. Taken together, the existing literature supports using common sociodemographic variables in exploratory subgroup analysis for the association between low social support and mental distress.

Preliminary research has shown insight by applying model-based recursive partitioning (MOB) for subgroup analysis in epidemiological studies with different designs and outcomes, indicating a promising use for subgroup discovery [16, 18–20]. MOB allows the potential discovery of higher-order interaction effects while avoiding prior specification of numerous interaction terms. Furthermore, conventionally specifying subgroups as higher-order interactions in a regression model becomes problematic as the amount of interaction terms grows large, presenting the problem of multiple comparisons. Additionally, MOB has an open-source implementation for the R statistical computing platform, which facilitates the analyses and further makes replication of our study more feasible for others. Comparing MOB to other similar techniques, the technique does not handle missing data [21], but imputation is performed to work around this limitation, as described further below. Comparing MOB to other subgroup analysis techniques, MOB has an advantage of simpler model interpretability over some other techniques, a well-known feature of tree models [21, 22]. MOB has not been previously used to study the association between social support and mental health, to identify new risk groups and heterogeneity in the association.

We thus examined the heterogeneity of the association between low social support and mental distress among old-age retired municipal sector employees of the City of Helsinki, Finland. The longitudinal cohort has a follow-up of more than 20 years. By using sociodemographic variables as effect modifiers in the analysis, MOB allows us to generate particular subgroups that are easy to interpret and contribute to policy formulation.

## Methods

### Data

**Fig. 1:**
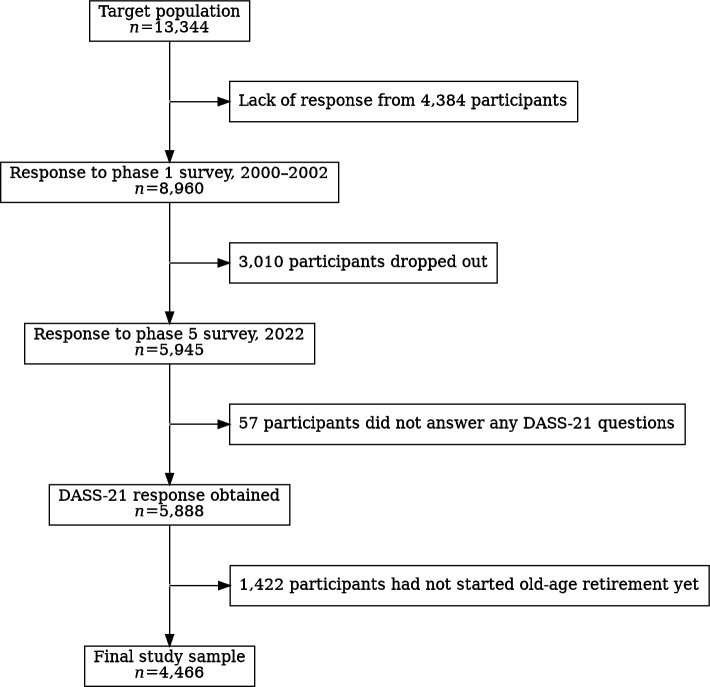
Flowchart of the participant inclusion process from the Helsinki Health Study, 2000–2022

The data were derived from the Helsinki Health Study (HHS) longitudinal cohort of current and former employees of the City of Helsinki, Finland [23]. The Phase 1 survey was mailed to employees reaching 40, 45, 50, 55, or 60 years of age during 2000, 2001, or 2002 (Fig. 1). During the Phase 5 survey in 2022, participants were 60 to 82 years old. The Phase 1 resulted in 8,960 participants (response rate 67%). Follow-up surveys were conducted in 2007 (Phase 2, response rate 83%), 2012 (Phase 3, response rate 79%), 2017 (Phase 4, response rate 82%), and 2022 (Phase 5, response rate 75%).

For this study, only participants who had started their old-age retirement before Phase 5 and answered at least some of the questions in the 21-question variant of the Depression Anxiety Stress Scales (DASS-21) questionnaires were included (*n* = 4,466). Earlier non-response analysis has showed that the survey data are broadly representative of the target population [24].

### Low social support

Low social support was operationalized by using a modified version of the number score (SSQN) of the Social Support Questionnaire (SSQ) [25], which measures the perceived quantity of social support contacts [26]. The questionnaire includes four of the 27 items from the original questionnaire, with the following wording in Finnish or Swedish: ”Whom can you really count on to help you feel more relaxed when you are under pressure or tension?”, ”Whom can you really count on to care about you regardless of what is happening to you?”, ”Whom can you really count on to help you feel better when you are feeling generally down-in-the-dumps?” and ”From whom do you get practical help when you need it?”. For each item, the participants were able to choose either “nobody” (zero score for the item) or one or more of the six categories of social contacts: A partner, a next of kin, a close friend, a co-worker or a supervisor, a neighbour, or another relative. Participants were able to choose each category only once. SSQN score was calculated by computing the mean of the number of contacts for the four items, and further aggregated by calculating the mean of the five study phases. The short four-item variant was used to reduce non-response bias, as the survey was lengthy and not planned to solely study social support and mental health but broad social and health-related questions.

The original version of SSQN has been shown to possess adequate test–retest reliability, factor validity, predictive validity, and concurrent validity [27]. SSQN measures the perceived quantity of social support, thus leaving out qualitative aspects of the construct.

To further evaluate the reliability and validity of the SSQN score within the modified SSQ instrument, we investigated the psychometric properties of the measure (please see the Supplementary Material, Table S1–Table S5). The SSQ item internal consistency was quantified with Cronbach’s alpha, (*α* = 0.90). The SSQ item intercorrelation matrix showed moderate correlation (Pearson’s *r*) between the items, as expected (Table S3). Individual frequencies for the items are displayed in Table S4, with no signs of the ceiling effect issue described with some SSQ variants. Finally, a regression analysis was conducted with other health-related quality of life outcomes to additionally evaluate the validity of the SSQ variant (Table S5).

### Mental distress

Mental distress was measured at Phase 5 in 2022 with the Depression Anxiety Stress Scales (DASS-21) with a common factor measure of general distress in addition to the three specific factors: Depression, anxiety, and stress [28]. The DASS-21 scales are not designed for psychiatric diagnosis, but they are found to correlate with the DSM-5 mood disorder and anxiety disorder symptoms over the past week. The DASS-21 depression scale has shown a high correlation (*r* = 0.79) [29] with the Beck Depression Inventory (BDI) [30]. Beck Anxiety Inventory (BAI) [31] correlation with DASS-21 anxiety scale was also high (*r* = 0.85). State-Trait Anxiety Inventory trait-scale [32] correlation with stress scale was moderate (*r* = 0.68) [29].

It was demonstrated in a systematic review of psychometric studies regarding DASS-21 that there is high-quality evidence that DASS-21 results typically have sufficient structural validity for a bifactor model [33]. Additionally, moderate-quality evidence is reported for sufficient content validity [33]. When evaluating DASS-21 against other similar measures, sufficient convergent validity was demonstrated [33]. Known-groups validity as well was sufficient, indicating that DASS-21 demonstrates adequate construct validity. The bifactor structure for DASS-21 was introduced after the original publication of DASS-21. Additionally, a study conducted in eight countries showed a better fit for the bifactor structure, demonstrating more consistent replication across cultures compared to the original three-factor structure [34]. A validation study of the bifactorial structure of DASS-21 is ongoing with this cohort of Finnish-speaking adults [unpublished]. Normative data from an Australian cohort with similar demographic characteristics were used to standardize the DASS-21 dimensions [35].

### Effect modifiers

Gender, age, marital status, and education were used as control variables and effect modifiers to form distinctive subgroups. Gender was self-reported with a binary question in Phase 1 during 2000–2002 (men vs. women).

Due to the stratified sampling of the data set, age was dichotomized into two categories as 60–72 and 73–82-year-olds. The first category mirrors the participants reaching 40, 45, and 50 years at Phase 1, while the second category mirrors employees reaching 55 and 60 years during Phase 1. The youngest age cluster (reaching 40 years of age during 2000–2002) was almost completely dropped from the study due to not reaching old-age retirements yet. In total, 1,422 participants were not yet on old-age retirement and were excluded from the study (Fig. 1).

Marital status was assessed in 2022, divided into three categories: Married, cohabiting or in a registered partnership; divorced, separated, or widowed; and unmarried. The Phase 5 marital status was chosen, as marital status may change during the follow-up, and marital status at Phase 5 is likely to be most closely linked to the outcome due to possible relevant changes in marital status of older adults, such as loss of a spouse.

Educational level was assessed only at Phase 1 and was categorised into three levels: High: Tertiary education (university degree or similar), Medium: Secondary education except vocational education (matriculation examination and college examination), Low: Compulsory education or vocational education. This categorisation groups similar levels of education together while distinguishing between different levels of education. Based on previous studies conducted using the same cohort, this categorisation predicts various health outcomes in line with the previous body of research and thus successfully operationalises educational attainment [36, 37].

### Missing data

Missing data were imputed using the R software package *mice* implementing multivariate imputation by the chained equations technique [38]. Altogether, 57 participants with completely empty DASS-21 questionnaires were excluded from the study. 296 participants answered at least one DASS-21 question, but not all, and were imputed. 24.7% of social support responses were partially or completely missing. The frequency of non-response for individual SSQ items is further detailed in Table S1, while the proportion of imputed data is clarified in Table S2 in the Supplementary Material. 1% of education responses and 2% of marital status responses were missing. Missing data were imputed using relevant modelling for each variable, such that variables with repeated measures were imputed with a random effects model with random intercepts.

### Statistical analysis

Risk factor heterogeneity was investigated with model-based recursive partitioning (MOB), an exploratory method integrating decision tree learning with regression analysis [16, 22].

MOB operates in an iterative fashion, partitioning the full sample into further subgroups by the following steps: (1) The given linear regression model is fitted to the dataset, and the candidate effect modifiers (partitioning variables) are specified for creating the subgroups. (2) Each effect modifier is tested for overall parameter instability. In other words, if the two resulting regression models differ significantly after division, instability is found. (3) If there is overall instability, the dataset is split with respect to the variable associated with the highest instability. (4) The procedure is repeated for each of the resulting subgroups until no more instability is found [22].

Thus, the procedure yields subgroups which can be graphically depicted as a tree, where the topmost split is the *primary effect modifier*, and further splits represent recursively the subsequent less important effect modifiers. Compared with a conventional decision tree approach which does not incorporate regression analysis, MOB has the advantage of providing adequate interpretability of risk factor associations with the outcome variables [39]. Compared with traditional regression methods with a priori interaction term specification, MOB provides a more parsimonious approach for interaction effect discovery, since fewer comparisons are required, especially for higher-order interactions. A variant of the MOB method, PALM tree (Partially Additive [Generalized] Linear Model Trees), was utilized to enable adjustment of the sociodemographic variables [40]. PALM trees implement global additive effects, which can be utilized to control for variables across the resulting subgroups. Tree size was limited by using a significance level of 1.0 (*alpha* parameter) and maximum depth of three (*maxdepth* parameter) for all subtrees, which should be appropriate for our exploratory analysis. This enables us to avoid very deep trees with small subgroups and excessively higher-order interactions, as maxdepth of three will limit them to three-way interactions.

The computational model was applied to the general distress factor and the three DASS-21-dimensions, using the same effect modifiers.

Analyses were performed in the *R* environment for statistical computing, using the *palmtree* software package (R version 4.1.3, palmtree version 0.9.1) [41].

## Results

### Characteristics of the study population

Table 1 displays characteristics of the study population. The majority (over 80%) were women, reflecting the female dominance of municipal sector workers. Some 45% of the participants were 73 years old or older, most (around 60%) were married or cohabiting and had low or medium education.

**Table 1:** Descriptive characteristics of the Helsinki Health Study participants 2000–2022

Characteristic	*N* = 4,466^a^
Gender	
Women	3,627 (81%)
Men	839 (19%)
Age (years, 2022)	
60–72	2,477 (55%)
73–82	1,989 (45%)
Marital status (2022)	
Unmarried	456 (10%)
Married or cohabiting	2,516 (58%)
Divorced or separated	1,390 (32%)
Education (2000–2002)	
Low	1,747 (39%)
Medium	1,360 (31%)
High	1,322 (30%)
Social support score (SSQN^c^, from 2000 until 2022), median (IQR)	1.50 (1.15, 2.05)
DASS-21^b^ General distress score, median (IQR)	5.7 (3.0, 9.0)
DASS-21 Depression score, median (IQR)	4 (2, 10)
DASS-21 Anxiety score, median (IQR)	4 (2, 6)
DASS-21 Stress score, median (IQR)	6 (2, 10)

^a^n (%); Median (IQR)

^b^DASS-21: Depression Anxiety Stress Scales

^c^SSQN: The Social Support Questionnaire

DASS-21 doubled mean (SD) scores were 5.97 (5.57) for general distress 6.39 (7.12) for depression, 4.90 (5.42) for anxiety and 6.86 (6.50) for stress. Depression and anxiety scales were to some degree higher, while stress scale was somewhat lower compared to other populations with similar demographics [35].

DASS-21 scales had zero score for 6.8% of participants for general distress, 22.9% for depression, 22.4% for anxiety and 19.3% for stress, implying no symptoms for the given dimension over the past week.

### Variable selection for DASS-21 effect modifiers

The tree is interpreted as a demographic division of subgroups, so that the topmost root node represents the full sample, while each child node represents a subgroup from their parent node. As the partitioning variables are chosen from a set of candidate variables with numerous tests, Bonferroni adjustment is used to mitigate the problem of multiple comparisons. The *p*-values compare two models fitted for the given subgroups, i.e., the equality for the given intercept and beta coefficient estimate for low social support.

**Fig. 2:**
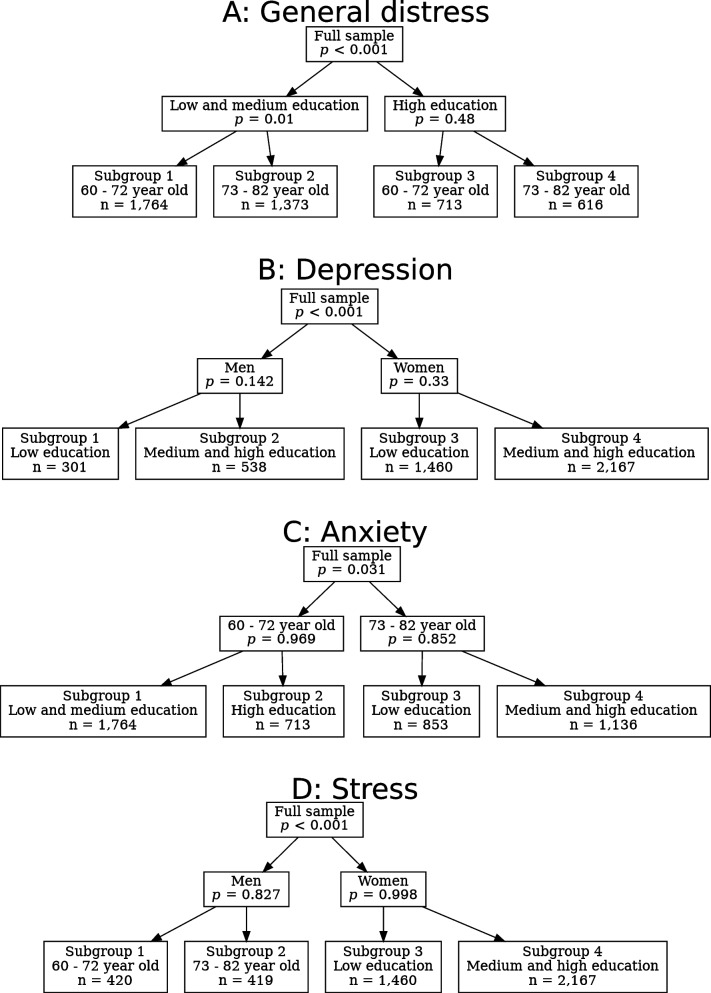
Model-based recursive partitioning (MOB)-trees of Depression Anxiety Stress Scales (DASS-21) for low social support, Helsinki Health Study (*n* = 4,466). The *p*-values represent the significance for difference between the two models with varying subgroups of people

For the general distress model, education is selected as the primary effect modifier, dividing the full sample into a low/medium education subgroup(non-tertiary education) and a high education subgroup (tertiary education). The anxiety model has less older retirees and older retirees in the first root partition. Depression and stress models have gender as the primary effect modifier (Fig. 2).

In the general distress model, more risk factor heterogeneity is detected compared to other models: In addition to the primary effect modifier of education, age is also a statistically significant effect modifier in the low and medium education subgroup.

### Risk factor estimates for mental distress

The coefficient estimates and confidence intervals for the associations between low social support and the mental distress dimensions are presented in Table 2. Even though the general distress model has two significant effect modifiers, education and age, the associations are fairly similar in all subgroups. In contrast, in the depression model the associations are notably heterogeneous, as men with medium and high education have the weakest association, while women with medium and high education have the strongest association. In the anxiety model, the participants with 73–82 years of age with low education have a stronger association, however the confidence intervals are large. Similarly, in the stress model men of 73–82 years of age have a stronger association, but the confidence interval is large.

**Table 2:** Estimates and 95% confidence intervals for z-score change in Depression Anxiety Stress Scales (DASS-21) dimensions for each decrease in social support score among old-age retirees (*n* = 4,466)

Subgroup	Coefficient estimate	95% CI
General distress^a^		
1: Low and medium education, 60–72 years of age	0.16	(0.12, 0.20)
2: Low and medium education, 73–82 years of age	0.15	(0.09, 0.20)
3: High education, 60–72 years of age	0.16	(0.10, 0.23)
4: High education, 73–82 years of age	0.19	(0.11, 0.26)
Depression^a^		
1: Men, low education	0.17	(0.02, 0.32)
2: Men, medium and high education	0.08	(-0.02, 0.17)
3: Women, low education	0.12	(0.05, 0.18)
4: Women, medium and high education	0.20	(0.15, 0.24)
Anxiety^a^		
1: 60–72 years of age, low and medium education	0.14	(0.09, 0.19)
2: 60–72 years of age, high education	0.10	(0.02, 0.17)
3: 73–82 years of age, low education	0.20	(0.11, 0.30)
4: 73–82 years of age, medium and high education	0.12	(0.06, 0.19)
Stress model^a^		
1: Men, 60–72 years of age	0.13	(0.02, 0.24)
2: Men, 73–82 years of age	0.23	(0.11, 0.35)
3: Women, low education	0.13	(0.06, 0.19)
4: Women, medium and high education	0.16	(0.11, 0.20)

^a^All models adjusted for gender, age, education, and marital status

## Discussion

### Main findings

Our study sought to produce new information about the role of social support in mental distress among old-age retired municipal sector employees applying a computational approach and examining several effect modifiers and higher-order interactions. Our findings highlight that gender, education and age are potential effect modifiers in the association of low social support and mental distress in old-age retirees. The model for depressive symptoms showed interactions for different educational levels, and education was the primary social support effect modifier for general distress. When education was medium or high, retired men showed notably weak association while retired women showed strong association of low social support and depressive symptoms.

### Interpretation

Our results are not directly comparable to previous studies on social support and mental distress, mainly due to different methodology as well as potential age, period, and cohort effects. Nonetheless, in cautious comparisons to earlier cohorts of older adults, our participants appear to have higher depression and anxiety scores, while stress scale scores were lower [35]. A possible explanation for these differences is that the scores reflect reduced work-related stress due to the retirement status of the participants. Previous studies have not used a similar computational approach (MOB) when examining the role of social support to mental health of older adults, or in any age group. Thus, confirmatory research is warranted before application of our results, although they verify the significance of low social support to mental distress in line with previous evidence [8, 9].

An advantage of the use of MOB is that it helps identify subgroups with heterogeneous associations for low social support. More specifically, examining effect modification using gender, age, marital status, and education provided new information and identified high-risk groups for potential intervention. While previous studies have shown that these sociodemographic variables act as effect modifiers in the association between social support and mental distress [12], our approach helped provide more in-depth and new information on the role of education and gender, suggesting higher-order interactions. Thus, the association between low social support and depressive symptom severity was different for women and men with the same level of education, with the association being strong for women with medium–high education but weak for men with medium–high education. In other words, with the exploratory approach inferring these subgroups with the most heterogeneous associations between low social support and mental distress, this approach produced a more detailed picture of the association. Comparing our results to similar studies with younger populations, a study with 32-year-old participants revealed a significant three-way interaction with gender, socioeconomic status and social support for the risk of depression. Among women, the effect of dissatisfaction with social support on the risk of depression was stronger in the manual than the non-manual class. This discrepancy with our results is possibly explained by age-specific differences in the phenomenon, or the differences in study design and measures [42]. These results suggest that merely adjusting for gender is insufficient when examining the associations between social support and mental health, or potentially other health outcomes among older people. The same approach can also be applied among younger people, and it could help identify new risk groups for earlier detection and intervention.

Finally, since there are apparent differences and higher-order interactions in the associations between social support and mental health, the disparities call for more careful consideration of gender and other effect modifiers in future studies, instead of assuming any uniform associations between risk factors and health outcomes.

This study additionally highlights the importance of considering differences between older adults of varying ages. The risk factor disparities are potentially shaped by differences in health conditions between recently retired and retirees approaching the final stages of their life course. As retirees age, they are increasingly likely to have cumulative health problems associated with ageing.

### Limitations and strengths

A key limitation is that we only had the DASS-21 measurement in the fifth study phase. Therefore, a random effects model, frequently used with longitudinal data, could not be applied in the main analysis, even though MOB is suitable for such modelling. Our study data set had a skewed gender distribution reflecting the target population [43]. Similarly, our sample excluded people outside the labour force at the study inclusion. The potentially conservative Bonferroni correction of the analysis is another possible limitation, increasing the risk of false negative errors for the tree model. Also, the confidence intervals of the estimates in Table 2 should be interpreted with caution due to the uncertainty in the statistical validity of the confidence intervals inferred after applying model selection through splitting into subgroups [16]. Finally, the chosen method produced one tree model which can be justified, however, it needs to be acknowledged that also other partitionings would have been possible.

A main strength of our study is the longitudinal study setting providing temporal sequence of changes, we thus had a follow-up of the same participants over a period of more than two decades and had measured social support identically in altogether five phases 2000–2022. Moreover, the outcome variable we used is validated and has been used in numerous previous studies. We also conducted validation of the social support measure used in this study (Supplementary Material, Table S1–Table S5). The results suggested that our measure possesses adequate psychometric properties in line with other brief SSQ variants. The data are large, which enabled addressing effect modifiers and higher order interactions, and applying a computational approach to provide new hypotheses and identify new risk groups in the research area. The retention of study participants was good throughout the study.

## Conclusions

Utilizing computational methods that provide automatic interaction discovery can be helpful when the studied question involves potential higher-order interaction effects. MOB, along with other exploratory methods, provide an adequate technique for subgroup analysis for observational epidemiology.

Our results suggest that among Finnish old-age retired municipal sector employees, low social support is a particularly critical risk factor for prevention and intervention among women with medium–high education. For studies investigating social support, stratification by gender and education or specifying higher-order interaction terms of these variables may provide valuable findings, confirming high-risk groups for policy implications and interventions.

## Supplementary Information

Supplementary Material 1.

## Data Availability

The authors confirm that, for approved reasons and due to Finnish data protection laws, access restrictions apply to the data underlying the findings and data cannot be shared. Requests and collaboration initiatives can be directed to the Helsinki Health Study research group (kttl-hhs@helsinki.fi).

## Abbreviations

BAI: Beck Anxiety Inventory
BDI: Beck Depression Inventory
CI: Confidence interval
DSM-5: Diagnostic and Statistical Manual of Mental Disorders, Fifth Edition
GDP: Gross domestic product
HHS: Helsinki Health Study
IQR: Interquartile range
MOB: Model-based recursive partitioning
OECD: Organisation for Economic Co-operation and Development
PALM tree: Partially Additive (Generalized) Linear Model tree
SD: Standard deviation
SSQ: Social Support Questionnaire (instrument)
SSQN: Social Support Questionnaire (number-subscale of the instrument)

## References

[1] Chang AY, Skirbekk VF, Tyrovolas S, Kassebaum NJ, Dieleman JL. Measuring population ageing: an analysis of the Global Burden of Disease Study 2017. Lancet Public Health. 2019;4:e159–67. 30851869 10.1016/S2468-2667(19)30019-2 PMC6472541

[2] OECD and European Union. Health at a Glance: Europe 2018: State of Health in the EU Cycle; 2018. 10.1787/health_glance_eur-2018-en.

[3] Wittchen HU, Jacobi F, Rehm J, Gustavsson A, Svensson M, Jönsson B, et al. The size and burden of mental disorders and other disorders of the brain in Europe 2010. Eur Neuropsychopharmacol. 2011;21:655–79. 21896369 10.1016/j.euroneuro.2011.07.018

[4] Pantell M, Rehkopf D, Jutte D, Syme SL, Balmes J, Adler N. Social Isolation: A Predictor of Mortality Comparable to Traditional Clinical Risk Factors. Am J Public Health. 2013;103:2056–62. 24028260 10.2105/AJPH.2013.301261 PMC3871270

[5] Klinenberg E. Social isolation, loneliness, and living alone: identifying the risks for public health. Am J Public Health. 2016;106:786–7. 27049414 10.2105/AJPH.2016.303166 PMC4985072

[6] Newman MG, Zainal NH. The value of maintaining social connections for mental health in older people. Lancet Public Health. 2020;5:e12–3. 31910976 10.1016/S2468-2667(19)30253-1 PMC7261393

[7] Santini ZI, Jose PE, York Cornwell E, Koyanagi A, Nielsen L, Hinrichsen C, et al. Social disconnectedness, perceived isolation, and symptoms of depression and anxiety among older Americans (NSHAP): a longitudinal mediation analysis. Lancet Public Health. 2020;5:e62-70. 31910981 10.1016/S2468-2667(19)30230-0

[8] Fiske A, Wetherell JL, Gatz M. Depression in older adults. Annu Rev Clin Psychol. 2009;5:363–89. 19327033 10.1146/annurev.clinpsy.032408.153621 PMC2852580

[9] Wolitzky-Taylor KB, Castriotta N, Lenze EJ, Stanley MA, Craske MG. Anxiety disorders in older adults: a comprehensive review. Depress Anxiety. 2010;27:190–211. 20099273 10.1002/da.20653

[10] Blazer DG, Hybels CF. Origins of depression in later life. Psychol Med. 2005;35:1241–52. 16168147 10.1017/S0033291705004411

[11] Hed S, Berg AI, Hansson I, Kivi M, Waern M. Gender differences in resources related to depressive symptoms during the early years of retirement: A Swedish population-based study. Int J Geriatr Psychiatry. 2020;35:1301–8. 32584479 10.1002/gps.5367

[12] Luo F, Guo L, Thapa A, Yu B. Social isolation and depression onset among middle-aged and older adults in China: Moderating effects of education and gender differences. J Affect Disord. 2021;283:71–6. 33524661 10.1016/j.jad.2021.01.022

[13] Gum AM, King-Kallimanis B, Kohn R. Prevalence of Mood, Anxiety, and Substance-Abuse Disorders for Older Americans in the National Comorbidity Survey-Replication. Am J Geriatr Psychiatry. 2009;17:769–81. 19700949 10.1097/JGP.0b013e3181ad4f5a

[14] Gariépy G, Honkaniemi H, Quesnel-Vallée A. Social support and protection from depression: systematic review of current findings in Western countries. Br J Psychiatry. 2016;209:284–93. 27445355 10.1192/bjp.bp.115.169094

[15] Colodro-Conde L, Couvy-Duchesne B, Zhu G, Coventry WL, Byrne EM, Gordon S, et al. A direct test of the diathesis–stress model for depression. Mol Psychiatry. 2018;23:1590–6. 28696435 10.1038/mp.2017.130 PMC5764823

[16] Seibold H, Zeileis A, Hothorn T. Model-based recursive partitioning for subgroup analyses. Int J Biostat. 2016;12:45–63. 27227717 10.1515/ijb-2015-0032

[17] Laaksonen E, Martikainen P, Lahelma E, Lallukka T, Rahkonen O, Head J, et al. Socioeconomic circumstances and common mental disorders among Finnish and British public sector employees: evidence from the Helsinki Health Study and the Whitehall II Study. Int J Epidemiol. 2007;36:776–86. 17517811 10.1093/ije/dym074

[18] Nguyen QC, Rehkopf DH, Schmidt NM, Osypuk TL. Heterogeneous effects of housing vouchers on the mental health of US Adolescents. Am J Public Health. 2016;106:755–62. 26794179 10.2105/AJPH.2015.303006 PMC4986050

[19] Pirkle CM, Wu YY, Zunzunegui M-V, Gómez JF. Model-based recursive partitioning to identify risk clusters for metabolic syndrome and its components: findings from the International Mobility in Aging Study. BMJ Open. 2018;8:e018680. 29500203 10.1136/bmjopen-2017-018680 PMC5855443

[20] Bowling CB, Berkowitz TSZ, Smith B, Whitson HE, DePasquale N, Wang V, et al. Unintended consequences of COVID-19 social distancing among older adults with kidney disease. J Gerontol. 2022;77:e133–7. 10.1093/gerona/glab211 PMC8344603 34286836

[21] Loh W, Cao L, Zhou P. Subgroup identification for precision medicine: A comparative review of 13 methods. WIREs Data Mining Knowl Discov. 2019;9:9.

[22] Zeileis A, Hothorn T, Hornik K. Model-Based Recursive Partitioning. J Comput Graph Stat. 2008;17:492–514.

[23] Lahelma E, Aittomäki A, Laaksonen M, Lallukka T, Martikainen P, Piha K, et al. Cohort profile: The Helsinki Health Study. Int J Epidemiol. 2013;42:722–30. 22467288 10.1093/ije/dys039

[24] Laaksonen M, Aittomäki A, Lallukka T, Rahkonen O, Saastamoinen P, Silventoinen K, et al. Register-based study among employees showed small nonparticipation bias in health surveys and check-ups. J Clin Epidemiol. 2008;61:900–6. 18486445 10.1016/j.jclinepi.2007.09.010

[25] Sarason IG, Levine HM, Basham RB, Sarason BR. Assessing social support: The social support questionnaire. J Pers Soc Psychol. 1983;44:127–39.

[26] Sarason BR, Shearin EN, Pierce GR, Sarason IG. Interrelations of social support measures: Theoretical and practical implications. J Pers Soc Psychol. 1987;52:813–32.

[27] Furukawa TA, Harai H, Hirai T, Kitamura T, Takahashi K. Social Support Questionnaire among psychiatric patients with various diagnoses and normal controls. Soc Psychiatry Psychiatr Epidemiol. 1999;34:216–22. 10365628 10.1007/s001270050136

[28] Lovibond PF, Lovibond SH. The structure of negative emotional states: Comparison of the Depression Anxiety Stress Scales (DASS) with the beck depression and anxiety inventories. Behav Res Ther. 1995;33:335–43. 7726811 10.1016/0005-7967(94)00075-u

[29] Antony MM, Bieling PJ, Cox BJ, Enns MW, Swinson RP. Psychometric properties of the 42-item and 21-item versions of the Depression Anxiety Stress Scales in clinical groups and a community sample. Psychol Assess. 1998;:176.

[30] Beck AT, Ward CH, Mendelson M, Mock J, Erbaugh J. An inventory for measuring depression. Arch Gen Psychiatry. 1961;4:561–71. 13688369 10.1001/archpsyc.1961.01710120031004

[31] Beck AT, Epstein N, Brown G, Steer RA. An inventory for measuring clinical anxiety: psychometric properties. J Consult Clin Psychol. 1988;56: 893. 3204199 10.1037//0022-006x.56.6.893

[32] Spielberger CD, Gonzalez-Reigosa F, Martinez-Urrutia A, Natalicio LFS, Natalicio DS. The state-trait anxiety inventory. Int J Psychol. 1971;5:145–58.

[33] Lee J, Lee E-H, Moon SH. Systematic review of the measurement properties of the Depression Anxiety Stress Scales–21 by applying updated COSMIN methodology. Qual Life Res. 2019;28:2325–39. 30937732 10.1007/s11136-019-02177-x

[34] Zanon C, Brenner RE, Baptista MN, Vogel DL, Rubin M, Al-Darmaki FR, et al. Examining the Dimensionality, Reliability, and Invariance of the Depression, Anxiety, and Stress Scale–21 (DASS-21) Across Eight Countries. Assessment. 2021;28:1531–44. 31916468 10.1177/1073191119887449

[35] Gomez R, Summers M, Summers A, Wolf A, Summers JJ. Depression Anxiety Stress Scales-21: Factor Structure and Test-Retest Invariance, and Temporal Stability and Uniqueness of Latent Factors in Older Adults. J Psychopathol Behav Assess. 2014;36:308–17.

[36] Salmela J, Kouvonen A, Mauramo E, Rahkonen O, Roos E, Lallukka T. Associations of childhood and adult socioeconomic circumstances with recommended food habits among young and midlife Finnish employees. BMC Nutr. 2022;8:65. 35836295 10.1186/s40795-022-00557-0 PMC9281257

[37] Mauramo E, Salmela J, Bogl LH, Lallukka T, Kanerva N. Multiple socioeconomic circumstances and trajectories of fruit and vegetable consumption: the Helsinki Health Study. Scand J Public Health. 2023;51:1144–52. 35535452 10.1177/14034948221094430 PMC11977821

[38] van Buuren S, Groothuis-Oudshoorn K. mice: Multivariate Imputation by Chained Equations in R. J Stat Softw. 2011;45:1–67.

[39] Rusch T, Zeileis A. Gaining insight with recursive partitioning of generalized linear models. J Stat Comput Simul. 2013;83:1301–15.

[40] Seibold H, Hothorn T, Zeileis A. Generalised linear model trees with global additive effects. Adv Data Anal Classif. 2019;13:703–25.

[41] R Core Team. R: A language and environment for statistical computing. Vienna: R Foundation for Statistical Computing; 2023.

[42] Huurre T, Eerola M, Rahkonen O, Aro H. Does social support affect the relationship between socioeconomic status and depression? A longitudinal study from adolescence to adulthood. J Affect Disord. 2007;100:55–64. 17070599 10.1016/j.jad.2006.09.019

[43] Martikainen P, Laaksonen M, Piha K, Lallukka T. Does survey non-response bias the association between occupational social class and health? Scand J Public Health. 2007;35:212–5. 17454926 10.1080/14034940600996563

