# Supplementary material for: Heterogeneity in the association between social support and mental distress in old-age retirees – a computational approach using longitudinal cohort data
Source: BMC Geriatr. 2024 Oct 8;24:813. doi: 10.1186/s12877-024-05384-5 (PMC11460196; doi:10.1186/s12877-024-05384-5)
Supplement: Supplementary file 1 — Supplementary Material 1. [file 12877_2024_5384_MOESM1_ESM.pdf]

Supplementary material: Validation of a four-item variant of the number-scale within Social Support Questionnaire (SSQ)

Contents

Supplementary material: Validation of a four-item variant of the number-scale within Social Support Questionnaire (SSQ) 1

Contents 1

Introduction........ 2

Table S1: SSQ data completeness for individual items across study phases 1 to 5 for the full Helsinki Health Study sample, among those who responded to each survey (n = 8,960)........ 4

Table S2: Proportion of imputed SSQ responses for individual items across study phases 1 to 5 for the subset of data used in the study (n = 4,466). 4

Table S3: Phase 5 (2022) SSQ item and overall score intercorrelations in the full Helsinki Health Study dataset (n = 5,787)........ 5

Table S4: SSQ item response distribution and descriptive statistics for phase 5 (n = 5,787)........ 5

Table S5: Regression analysis of SSQN and RAND-36 health scales across study phases 1 (2000–2002) to 5 (2022) (n = 8,960)........ 6

References 6

Introduction

We evaluated the psychometric properties of a four-item social support measure based on the number-subscale (SSQN) of the Social Support Questionnaire (SSQ) [1]. Our variant includes the following items from the original SSQ: 1. ”Whom can you really count on to help you feel more relaxed when you are under pressure or tension?“, 2. "Whom can you really count on to care about you regardless of what is happening to you?“, 3. ”Whom can you really count on to help you feel better when you are feeling generally down-in-the-dumps?" and 4. ”From whom do you get practical help when you need it?“. Instead of asking the participants to list all available support contacts for each category, we provided six distinct types of typical social support contacts, in addition to the "nobody"-option. The six types are as follows: A partner, a next of kin, a close friend, a co-worker or supervisor, a neighbour, or another relative. Participants select one or more options, so that the resulting score for each item ranges from zero to six. The four items are summed, and then the mean is calculated to determine the SSQN score in line with the original 27-item SSQN.

We analysed the missing data over the five study phases and four SSQ items and show how individual items are imputed (Table S1 and Table S2). Overall, 24.7% of study participants had one or more SSQ items missing over any of the study phases (Table S2). Details about the cohort’s characteristics are provided in a previous publication [2]. The study data subset inclusion criteria are detailed in the *Methods* section and Figure 1. Complete SSQ responses were more often obtained in phases 1 and 5, as our data subset only included those who completed the survey in these phases. Complete response for all phases and SSQ items were obtained from 75.3% of participants in the study sample (n = 4,466).

We calculated the intercorrelations between the items and the overall SSQN score, presented in Table S3. A moderate correlation was observed between the items, suggesting similar underlying construct but no indication of item redundancy.

Item response frequencies and descriptive statistics are presented in Table S4. We found no evidence of the ceiling effect issue reported for other brief SSQ variants [3]. The mean (SD) for phase 5 SSQN was 1.68 (0.82) based on the full sample (n = 5,787). Skewness and excess kurtosis were 0.91 and 0.60, respectively, while Cronbach’s alpha was 0.90.

Finally, a linear random effects model was fitted to the longitudinal responses from Phase 1 (2000) to Phase 5 (2022) to investigate the association between SSQN and RAND-36 general health statuses, shown in Table S5. The random effect included an intercept for each participant, with three models fitted for each dependent variable with varying adjustments. RAND-36 is a general health status questionnaire comprising seven scales,

where scores range from 0 to 100 [4]. Prior studies have investigated the validity of RAND-36 measures in the Finnish population along with nationally representative descriptive statistics stratified by age and gender, as shown in Tables 10 to 12 in the publication by Aalto et al. [5]. Three models were fitted: One with crude associations; one adjusted for gender, age, and education; and a fully adjusted model which also included marital status. One additional social support contact was associated with an increment of 1.67 in RAND-36 General Health score (Table S5). The analysis suggests our SSQ variant for measuring social support is associated with constructs of mental distress similarly to the original SSQ instrument [6].

Table S1: SSQ data completeness for individual items across study phases 1 to 5 for the full Helsinki Health Study sample, among those who responded to each survey (n = 8,960).

	SSQ item			
**Study phase[1]**	1	2	3	4
**1 (2000-2002, n = 8,960)**	98.9%	98.8%	98.5%	98.7%
**2 (2007, n = 7,332)**	99.1%	98.9%	98.5%	98.8%
**3 (2012, n = 6,814)**	98.6%	98.8%	98.3%	98.5%
**4 (2017, n = 6,832)**	98.5%	98.7%	98.2%	98.6%
**5 (2022, n = 5,945)**	98.0%	98.0%	97.6%	97.9%

Table S2: Proportion of imputed SSQ responses for individual items across study phases 1 to 5 for the subset of data used in the study (n = 4,466).

	SSQ item			
**Study phase[1]**	1	2	3	4
**1 (2000-2002)**	0.8%	0.9%	1.3%	1.0%
**2 (2007)**	9.3%	9.3%	9.7%	9.4%
**3 (2012)**	10.9%	10.7%	11.1%	10.8%
**4 (2017)**	7.0%	6.8%	7.4%	6.9%
**5 (2022)**	1.9%	1.9%	2.3%	2.0%

[1]All values reported as proportion of imputed item response in relation to the study participants. Due to the study subsample inclusion criteria (Figure 1), a higher proportion of imputed data during phases 2-4 is expected.

Table S3: Phase 5 (2022) SSQ item and overall score intercorrelations in the full Helsinki Health Study dataset (n = 5,787)

	SSQ Item			
**SSQ Item**[1]	2	3	4	Overall SSQN
**1**	0.72	0.70	0.67	0.88
**2**	-	0.72	0.72	0.90
**3**	-	-	0.65	0.87
**4**	-	-	-	0.87

[1]All reported values are Pearson's *r*.

Table S4: SSQ item response distribution and descriptive statistics for phase 5 (n = 5,787)

	Item			
**Characteristic**	1	2	3	4
**Score frequency**[1]				
0	4.0%	1.2%	4.7%	1.7%
1	52.6%	45.1%	53.3%	48.4%
2	27.2%	31.3%	25.9%	29.3%
3	12.6%	17.2%	13.3%	14.2%
4	3.0%	4.3%	2.5%	5.1%
5	0.4%	0.8%	0.3%	1.1%
6	0.1%	0.1%	0.1%	0.1%
**Mean (SD)**	1.6 (0.9)	1.8 (0.9)	1.6 (0.9)	1.8 (1.0)
**Skewness**	1.0	0.9	0.9	1.1
**Kurtosis**[2]	1.1	0.4	0.8	0.8

[1]SSQ Number score representing quantity of social support contacts.

[2]Reported kurtosis values are excess kurtoses.

Table S5: Regression analysis of SSQN and RAND-36 health scales across study phases 1 (2000–2002) to 5 (2022) (n = 8,960).

Outcome	Model[1]	Beta[2]	95% CI	AIC
**General health perceptions**	1	2.05	1.83, 2.27	299182
	2	1.86	1.64, 2.08	296344
	3	1.74	1.52, 1.97	293870
**Physical functioning**	1	1.63	1.41, 1.85	296720
	2	1.29	1.07, 1.51	293311
	3	1.11	0.88, 1.33	290725
**Emotional well-being**	1	2.23	2.05, 2.42	282649
	2	2.35	2.17, 2.54	280160
	3	2.27	2.09, 2.46	278034
**Social functioning**	1	2.46	2.21, 2.72	309416
	2	2.49	2.23, 2.75	306651
	3	2.38	2.12, 2.65	304135
**Energy**	1	1.99	1.76, 2.21	296096
	2	2.17	1.94, 2.39	293481
	3	2.15	1.92, 2.37	291261
**Bodily pain**	1	1.02	0.76, 1.28	307798
	2	0.85	0.59, 1.11	304747
	3	0.86	0.59, 1.12	302393
**Role functioning: physical**	1	3.04	2.60, 3.49	345603
	2	2.48	2.04, 2.93	342191
	3	2.29	1.83, 2.74	339396
**Role functioning: emotional**	1	4.16	3.75, 4.58	340674
	2	3.87	3.45, 4.29	337570
	3	3.57	3.14, 3.99	334749

[1]Model 1: Crude unadjusted model. Model 2: Adjusted for age, gender and education. Model 3: Adjusted for variables in model 2 and marital status.

[2]Unstandardized beta coefficients.

References

1. Sarason IG, Levine HM, Basham RB, Sarason BR. Assessing social support: The Social Support Questionnaire. J Pers Soc Psychol. 1983;44:127–39.

2. Lahelma E, Aittomäki A, Laaksonen M, Lallukka T, Martikainen P, Piha K, et al. Cohort Profile: The Helsinki Health Study. Int J Epidemiol. 2013;42:722–30.

3. Sarason IG, Sarason BR, Shearin EN, Pierce GR. A Brief Measure of Social Support: Practical and Theoretical Implications. J Soc Pers Relat. 1987;4:497–510.

4. Hays RD, Sherbourne CD, Mazel RM. The rand 36-item health survey 1.0. Health Econ. 1993;2:217–27.

5. Aalto A-M, Aro AR, Teperi J. Rand-36 terveyteen liittyvän elämänlaadun mittarina : Mittarin luotettavuus ja suomalaiset väestöarvot. 1999.

6. Sarason BR, Shearin EN, Pierce GR, Sarason IG. Interrelations of social support measures: Theoretical and practical implications. J Pers Soc Psychol. 1987;52:813–32.
